# Self-Reported Hearing/Visual Loss and Mortality in Middle-Aged and Older Adults: Findings From the Komo-Ise Cohort, Japan

**DOI:** 10.2188/jea.JE20180198

**Published:** 2020-02-05

**Authors:** Atsushi Miyawaki, Yasuki Kobayashi, Ichiro Kawachi

**Affiliations:** 1 Department of Public Health, Graduate School of Medicine, The University of Tokyo, Tokyo, Japan; 2 Department of Social and Behavioral Sciences, Harvard T.H. Chan School of Public Health, Boston, MA, United States

**Keywords:** sensory loss, mortality, survival analysis, mediation analysis, Japan

## Abstract

**Background:**

The association of sensory loss with mortality remains unclear. We aimed to explore the associations of hearing loss (HL), visual loss (VL), and dual sensory loss (DSL) with survival.

**Methods:**

Data came from the *Komo-Ise* study cohort in Gunma Prefecture, Japan, where the community-dwelling residents aged 40–69 years were followed up from 1993 to 2010. We analyzed 9,522 individuals who answered the follow-up questionnaires in 2000 (average age 64 [range, 47 to 77] years in 2000). The primary exposures were “HL only,” “VL only,” or “DSL”, with “no HL/VL” as the reference. These sensory loss statuses were assessed by asking the difficulty in hearing conversation or reading newspaper even with aids in the follow-up questionnaires in 2000. All-cause and cause-specific mortality were ascertained from linkage to death certificate data. Cox proportional hazards models adjusting for confounders, including demographic factors, socioeconomic status, and health status, were used. Potential mediators (depression, walking disability, and social participation) were additionally adjusted for.

**Results:**

There were 1,105 deaths over the 10-year follow-up. After adjustment for the potential confounders, HL and DSL were associated with increased all-cause mortality (hazard ratios of 1.74 [95% CI, 1.18–2.57] and 1.63 [95% CI, 1.09–2.42], respectively). Potential mediators explained a modest portion of the association. As for cause-specific mortality, HL was associated with increased cancer mortality, while VL and DSL were associated with increased cardiovascular disease mortality.

**Conclusions:**

Self-reported HL and DSL may be risk factors of mortality among middle-aged or elderly Japanese populations.

## INTRODUCTION

Sensory loss, such as hearing loss (HL) or visual loss (VL), is commonly observed with advancing age. Sensory loss restricts communication with others (including doctor-patient communication) and may inhibit social participation, leading to declining physical activity,^1^ psychological health,^2^^,^^3^ as well as cognitive function,^1^ all of which have been suggested to be risk factors for premature mortality in previous studies.^4^^–^^6^ The association of sensory loss with all-cause mortality and cardiovascular disease (CVD) mortality has been investigated,^7^^–^^15^ but with conflicting results. Significant associations with increased mortality were observed after adjustment for potential covariates, including CVD risk factors, in some studies of HL^7^^,^^8^^,^^10^ or of VL,^11^^,^^12^ but not in other studies of HL^9^^,^^13^^,^^15^ or VL.^13^^–^^15^ These inconsistencies may be partly due to residual confounding by failure to mutually adjust for HL and VL.^13^ It is important to consider HL and VL simultaneously because they have common risk factors (eg, a history of smoking) and often occur together rather than independently.

However, in most prior studies that investigated the association of sensory loss and mortality, HL^7^^–^^10^ or VL^11^^,^^12^ was considered singly, not together. A limited number of studies have addressed HL and VL simultaneously with sufficient adjustment for potential confounders. Schubert et al,^13^ Gopinath et al,^14^ and Fisher et al^15^ explored the association of sensory loss, including HL/VL, with survival, assessed using objective hearing and vision examinations, such as audiometric assessments and best corrected visual acuity tests. In these studies, combined HL and VL were associated with increased mortality. On the other hand, the association between *subjective* sensory loss in daily life (measured using self-report) should be addressed, because: (a) even if people have the same level of sensory loss as assessed via objective examinations, their symptoms may be different depending on the environmental context, the causes of the loss, and coping resources,^16^ and (b) assessing subjective sensory loss does not require measurement devices used in audiometry and visual acuity testing. In the present study, therefore, we aimed to test the association between self-reported sensory loss and mortality with adjustment for as many potential confounders as possible.

## MATERIAL AND METHODS

### Data

Data was obtained from the *Komo-Ise* study cohort in Gunma Prefecture in the Kanto region of Japan.^17^^–^^20^ The *Komo-Ise* study was established in 1993, when self-administered questionnaires were distributed to all the inhabitants aged 40–69 years in Komochi village (rural area, *n* = 4,875) and a downtown area of Isesaki city (urban area, *n* = 7,755) through the local municipal offices. The first wave of the survey in 1993 queried information on demographics, socioeconomic status (SES), lifestyles, and health-related factors, and 11,565 residents responded (response rate: 91.6%). In 2000, follow-up questionnaires (a Japanese version of the Alameda County Study 1999 questionnaires) were distributed through the local municipal offices to the 10,898 surviving and uncensored participants who responded in 1993, and 9,650 participants responded (response rate: 88.5%).^20^ Among these, 9,522 participants who still lived in the study areas as of November 1, 2000 (which we set as the start of the observation period) were analyzed. The participants were followed up until 2010. Missing information on key variables were imputed under fully conditional specification using multiple imputations by chained equations.^21^ The imputation model was specified for an exposure, covariates, and outcomes using multinomial logistic regression (only categorical variables were missing). Ten datasets were generated, and the results were pooled using Rubin’s rules.^21^ The percentages of missing values for the key variables ranged from 0% to 15.2% (eTable 1); and 33.3% of the analytic participants had at least one missing key variable. This study was approved by the institutional Review Board of the University of Tokyo (approval no. 11153) and was conducted adhering to the tenets of the Declaration of Helsinki. Written informed consent was obtained from all the respondents at the beginning of the study.

### Sensory loss

The participants were categorized into four groups according to self-reported sensory loss: 1) no HL/VL, 2) HL only, 3) VL only, and 4) dual sensory loss (DSL). The dummies of HL only, VL only, and DSL were jointly included as primary exposures with no HL/VL as the reference. The participants were asked in 2000: “How much difficulty do you have hearing and understanding words in a normal conversation (even with a hearing aid)?” and “How much difficulty do you have seeing well enough to read a newspaper (even with glasses)?”. Response sets used for assessing the level of hearing or visual difficulty were “a great deal,” “some,” “a little,” or “none.” The participants were considered as having HL or VL when they answered “a great deal” or “some” for each question. These question sets were previously used in Alameda County Study.^22^

### Mortality and censoring

Deaths were identified through linkage to death certificate data recorded in Japan’s compulsory registration system of the study areas from November 1, 2000, to October 1, 2010 (study period). Participants who had migrated out of the study areas were contacted by mail, and non-responders were censored. The cause of death was recorded using the International Classification of Diseases 10th edition.^23^ Cancer death (C00–C97 and D00–D48) and cardiovascular disease (CVD) death (I00–I99) were identified.

### Covariates

The following covariates were adjusted for in accordance with the previous studies^13^^,^^15^: demographic factors (sex, age, marital status, and the living area); SES (education level, income level, and the job category); health status (self-rated health, self-reported medical history, and body mass index [BMI]); and health-related behaviors (smoking status, exercise habits, alcohol consumption, and dietary patterns). Racial variation was minimal in this Japanese setting and therefore ignorable. Age was categorized into six groups: 47–51, 52–56, 57–61, 62–66, 67–71, and 72–77 years. Marital status was categorized into three groups: married, separated/divorced, and never married. Education level was categorized into three groups according to education years: 9 or fewer years, 10–15 years, and ≥16 years. We added a fixed effect for Isesaki city, with residence in Komochi village as the reference. Three income-level categories were established according to tertiles of calculated household equivalized (pre-tax) income. The primary occupation, which was answered in the questionnaires distributed in 1993, was categorized into six groups: “not employed,” “agricultural/forestry,” “self-employed,” “blue-collar worker,” “white-collar worker,” and “others”. Self-rated health was dichotomized (1 = “Very good,” “Good,” or “Fair” and 0 = “Bad”). Self-reported medical histories were asked concerning cancer, stroke, heart disease, diabetes, dyslipidemia, and hypertension, respectively. BMI was calculated by dividing self-reported body weight (kg) by self-reported body height squared (m^2^). Health-related behaviors were defined as follows: smoking status (never smoker, past smoker, or current smoker), exercise habits (1 if a participant does an exercise “sometimes” or “often”, and 0 otherwise), alcohol consumption pattern (non-drinker, slight/moderate drinker [0–40 g/day for men and 0–20 g/day for women], or heavy drinker [>40 g/day for men and >20 g/day for women]).^24^ Previous studies consistently reported the association of smoking status with HL^25^ and VL,^26^ while the association of alcohol consumption pattern was not clearly defined; heavy alcohol consumption may decline hearing/visual function,^27^^,^^28^ but moderate consumption may prevent HL/VL.^28^^,^^29^ Dietary patterns, which is reportedly associated with HL^30^ or VL,^31^ were asked concerning the dichotomous preference of salty foods, sweet foods, or fatty foods. All the covariates were assessed based on the questionnaires distributed in 2000 except for education level and primary occupations, which were assessed in 1993.

### Potential mediators

As potential mediators, indicators of depression, walking disability, and social participation were considered.^7^^,^^11^ That is, if sensory loss is correlated with increased risk of mortality, the association may be mediated by these variables, because: (a) hearing/vision loss is likely to result in depression, walking disability, and decreased social participation,^1^^,^^3^ and (b) depression, ambulatory disability, and decreased social participation are each associated with premature mortality.^5^^,^^6^^,^^32^ Depression was defined following the previous study^22^ using a set of 12 items that operationalized the diagnostic criteria for a major depressive episode outlined in Diagnostic and Statistical Manual of Mental Disorders.^33^ Walking disability was dichotomized (0 = “no difficulty,” and 1 = “some difficulty,” “a lot of difficulties,” or “cannot do it without help”), according to the question “How difficult is it for you to walk across a room?”. The participants who chose “Often” or “Sometimes” for at least one of the three following questions were considered to have social participation: “How often do you visit with family or friends?”, “How often do you go out for community or volunteer activities?”, and “How often do you participate in hobby or community clubs?” (they were asked to choose one of “Often”, “Sometimes”, or “Never.”).

### Statistical analyses

In the main analyses, Cox proportional hazards models were applied. Sequential statistical adjustments were conducted: in model 1, age and sex were adjusted; in model 2, the other demographic factors and SES were additionally adjusted; and in model 3, health status and health-related behaviors were additionally adjusted. Using model 3, we also conducted analyses including sex/age (≥62 years old or not) x sensory loss statuses interaction terms, because the effect of sensory loss on mortality might differ by age and sex (for example, the effects of sensory loss on social/health factors might depend on sex and age).^15^^,^^34^ Furthermore, the potential mediators mentioned above were added to model 3 separately or jointly. The analyses were repeated for cancer mortality and CVD mortality as outcomes. In contrast to the previous studies,^15^ we focused on cancer mortality as well as CVD mortality, because 1) these are leading causes of death in Japan (28.5% and 23.5% of all-cause deaths in 2016 in the whole of Japan, respectively),^35^ and 2) we supposed that the decreased social participation derived from sensory loss might increase cancer mortality.^36^ Robust standard errors were calculated. Two-tailed *P* values below 0.05 were interpreted as statistically significant. The proportional hazards assumption was evaluated graphically and via Schoenfeld test; there was no violation of the assumption for each model. These analyses were conducted using Stata 15 (Stata Corp., College Station, TX, USA).^37^

### Sensitivity analyses

First, we repeated the main analyses among participants with no missing key variables (ie, listwise deletion; *n* = 6,349). Second, we repeated the analyses using stricter definitions of sensory loss, where the participants were considered as having HL or VL when they answered “a great deal.” Third, for the exposure(s) associated with all-cause mortality in model 3, we applied mediation analyses using Aalen additive hazards model (eAppendix 1) and quantified the percentage of the indirect effect contributed by each potential mediator to the total effect, following Lange and Hansen^38^ using R 3.5.0 (R Development Core Team, Vienna, Austria).^39^ Fourth, we conducted the analyses that separately included HL/VL in model 3 following the prior studies where HL or VL was considered singly.^7^^–^^12^

## RESULTS

Table 1 shows descriptive statistics of the participants according to type of sensory loss. Compared to those who had no HL/VL, those who had HL only, VL only, or DSL were older; less educated; less likely to be married; more likely to report lower income; more likely to have a history of cancer, stroke, heart disease, diabetes, or hypertension; more likely to report walking disability; more depressed; and less involved in social participation. The average follow-up period was 9.0 years. Among the 9,522 participants, 1,105 people (11.6%) died over the 10-year follow-up. Only 36 individuals (0.4%) were lost to follow-up.

**Table 1.** Descriptive statistics of the analytic participants

Characteristics		No hearing/visual loss	Hearing loss only	Visual loss only	Dual sensory loss	Missing information on hearing or visual loss
Number of participants		8074	87	636	86	639
Person-years at risk		73484	736	5578	678	5660
Men		47.0	57.5	44.8	52.3	46.0
Age, mean (SD)		61.5 (8.2)	67.6 (7.2)	63.1 (8.2)	68.2 (7.2)	65.7 (7.8)
Hearing loss only		0.0	100.0	0.0	0.0	12.9
Visual loss only		0.0	0.0	100.0	0.0	24.4
Dual sensory loss		0.0	0.0	0.0	100.0	0.0
Education years	*≧16+ years*	11.2	6.0	11.4	0.0	3.9
	*10 to 15 years*	47.9	28.6	43.0	32.5	27.4
	*≦9 years*	40.9	65.5	45.6	67.5	68.7
Income level	*Low*	31.2	32.1	37.6	59.2	57.1
	*Intermediate*	34.0	37.0	34.5	23.7	24.4
	*High*	34.8	30.9	27.9	17.1	18.5
Living area	*Urban*	59.8	57.5	60.7	48.8	48.0
	*Rural*	40.2	42.5	39.3	51.2	52.0
Marital status	*Never married*	5.0	9.1	5.4	3.8	4.0
	*Separated/divorced*	13.1	18.2	16.5	25.3	16.8
	*Married*	81.9	72.7	78.2	70.9	79.2
Primary occupation	*Unemployed*	3.9	6.9	7.5	8.1	3.9
	*Farmer/forestry worker*	15.3	20.7	19.0	10.5	15.3
	*Self-employed*	48.7	44.8	47.0	60.5	48.7
	*Blue collar*	7.4	9.2	8.2	9.3	7.4
	*White collar*	19.3	13.8	13.7	8.1	19.3
	*Others*	5.4	4.6	4.6	3.5	5.4
Exercise habits		45.3	48.6	34.5	29.0	42.2
Alcohol consumption	*Light/moderate*	49.4	61.2	44.2	33.3	45.3
	*Heavy*	7.0	5.9	7.4	7.4	8.0
Smoking status	*Current smoker*	28.7	29.6	29.1	36.1	37.3
	*Ever smoker*	19.4	28.4	20.6	22.2	21.3
Body mass index >25		23.6	15.7	26.5	29.3	23.1
Dietary patterns (preference for)	*Salty foods*	13.5	12.9	18.2	18.5	12.3
	*Sweet foods*	31.6	36.5	34.1	37.0	30.4
	*Fatty foods*	17.7	24.1	21.1	20.5	16.2
Fair or better self-rated health		97.1	96.6	87.3	74.1	97.1
History of cancer		3.7	6.9	5.5	5.8	2.8
History of stroke		3.2	4.6	9.1	19.8	3.2
History of heart disease		12.1	27.6	17.5	20.9	12.1
History of diabetes		8.4	10.3	13.5	15.1	8.4
History of dyslipidemia		20.4	16.1	20.3	22.1	20.4
History of hypertension		29.3	36.8	34.4	36.0	29.3
Depression		1.8	5.7	5.7	19.0	4.7
Walking disability		3.7	8.6	17.2	37.7	10.8
Social participation		84.5	74.4	72.8	58.2	84.5
Death	*All-cause*	10.4	26.4	16.0	34.9	17.5
	*Cancer*	3.5	10.3	3.9	11.6	4.9
	*CVD*	2.0	3.4	5.3	16.3	4.5
	*Others*	4.9	12.7	6.8	7.0	8.1

CVD, cardiovascular disease; SD, standard deviation.

Dual sensory loss was defined as having both hearing loss and visual loss. History of cancer, stroke, heart diseases, diabetes, dyslipidemia, and hypertension indicate self-reported history. Primary occupation was recorded in the questionnaire distributed in 1993. Percentages are calculated except for the number of the participants, age and person-years at risk within the sensory loss category.

**Figure 1.**
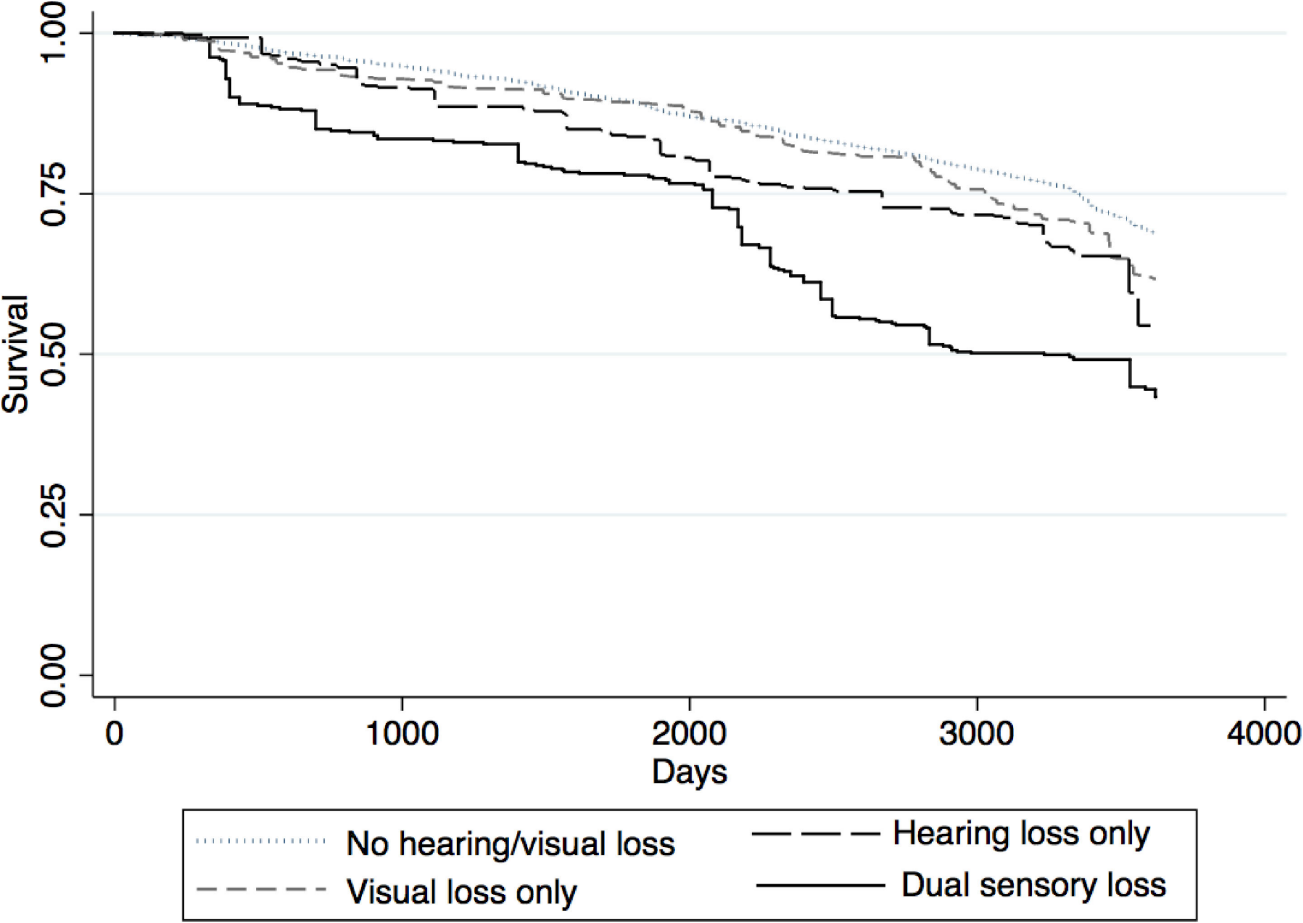
Age- and sex-adjusted survival curves for each status of the sensory loss.

Table 2 reports the association between self-reported sensory loss and all-cause mortality. The covariates were sequentially adjusted for (Panel A). Those who had HL only, VL only, or DSL showed a higher risk of early death compared to those who had no HL/VL after adjusting for age and sex (model 1: hazard ratio [HR] 1.72; 95% confidence interval [CI], 1.16–2.55, HR 1.48; 95% CI, 1.20–1.83, or HR 2.51; 95% CI, 1.68–3.74, respectively). Figure 1 displays the age- and sex-adjusted survival curves for each status of the sensory loss. In model 2 (adjusted for demographic factors and SES), the associations remained similar. Finally, after adjustment for the health status and health-related behaviors (model 3), the association of HL or DSL with mortality remained statistically significant (HR 1.74; 95% CI, 1.18–2.57 and HR 1.63; 95% CI, 1.09–2.42, respectively). Meanwhile, the association between VL only and mortality was attenuated and became statistically insignificant (HR 1.05; 95% CI, 0.83–1.32). The interaction terms of sex x HL only, VL only, and DSL for all-cause mortality were all insignificant (*P* = 0.81, *P* = 0.61, and *P* = 0.32, respectively). The interaction terms of older group (aged ≥62 years) x HL only, VL only, and DSL were all insignificant (*P* = 0.77, *P* = 0.55, and *P* = 0.44, respectively).

**Table 2.** The association between sensory loss status and all-cause mortality

	Hazard ratios (95% confidence intervals)		

	Hearing loss only	Visual loss only	Dual sensory loss
*A: Adjustment for covariates*			
Model 1	1.72^**^ (1.16, 2.55)	1.48^***^ (1.20, 1.83)	2.51^***^ (1.68, 3.674)
Model 2	1.71^**^ (1.16, 2.53)	1.42^***^ (1.15, 1.76)	2.45^***^ (1.65, 3.64)
Model 3	1.74^**^ (1.18, 2.57)	1.05 (0.83, 1.32)	1.63^*^ (1.09, 2.42)
*B: Adjustment for potential mediators*			
Model 3 + Depression	1.71^**^ (1.15, 2.55)	1.04 (0.82, 1.31)	1.55^*^ (1.03, 2.34)
Model 3 + Walking disability	1.62^*^ (1.10, 2.40)	0.95 (0.75, 1.20)	1.36 (0.90, 2.08)
Model 3 + Social participation	1.72^*^ (1.16, 2.53)	1.01 (0.80, 1.28)	1.58^*^ (1.07, 2.34)
Model 3 + All potential mediators	1.60^*^ (1.08, 2.37)	0.93 (0.73, 1.17)	1.31 (0.85, 2.00)

A Cox proportional hazards model was applied. In Model 1, age and sex were adjusted; in Model 2, the other demographic factors (education years, the living area, income level, marital status, and primary occupation) were additionally adjusted; and in Model 3, health statuses and health behaviors (self-rated health, self-reported histories of cancer, stroke, heart disease, diabetes, dyslipidemia, and hypertension, body mass index, smoking status, exercise habits, alcohol consumption, and dietary patterns) were additionally adjusted. In Panel B, the indicators of walking ability, depression, and social participation were separately or jointly adjusted as potential mediators. The reference group was participants without hearing loss or visual loss.

^*^P < 0.05. ^***^P < 0.001.

Panel B in Table 2 shows that the associations between HL and mortality remained significant after adjustment for each of the mediators, while the significant association between DSL and mortality disappeared after adjustment for walking disability. In the mediation analysis, the percentages of the indirect effect relative to the total effect in the association of HL only with all-cause mortality were 2.4% for depression, 1.6% for walking disability, and 3.6% for social participation. The percentage of the indirect effect relative to the total effect in the association of DSL with all-cause mortality were 4.0% for depression, 23.8% for walking disability, and 2.6% for social participation. The statistical significance was not observed.

Table 3 shows the associations between self-reported sensory loss and cause-specific mortality. HL was significantly associated with cancer mortality after adjustment for all potential covariates (HR 2.19; 95% CI, 1.16–4.12). DSL was significantly associated with CVD mortality after adjustment for all the potential covariates (HR 2.82; 95% CI, 1.46–5.45). After adjustment for each of the potential mediators, the association of HL with cancer mortality and the association of DSL with CVD mortality remained significant.

**Table 3.** The association between sensory loss and cause-specific mortality

	Hazard ratios (95% confidence intervals)		

	Hearing loss only	Visual loss only	Dual sensory loss
Cancer mortality			
*A: Adjustment for covariates*			
Model 1	2.02^*^ (1.08, 3.77)	1.12 (0.74, 1.70)	2.55^**^ (1.35, 4.82)
Model 2	2.10^*^ (1.12, 3.95)	1.09 (0.72, 1.66)	2.69^**^ (1.41, 5.13)
Model 3	2.19^*^ (1.16, 4.12)	0.86 (0.56, 1.33)	1.95 (0.98, 3.85)
*B: Adjustment for potential mediators*			
Model 3 + Depression	2.11^*^ (1.12, 4.00)	0.85 (0.55, 1.32)	1.79 (0.88, 3.61)
Model 3 + Walking disability	2.15^*^ (1.14, 4.06)	0.84 (0.54, 1.30)	1.84 (0.91, 3.72)
Model 3 + Social networks	2.18^*^ (1.16, 4.10)	0.85 (0.55, 1.33)	1.93 (0.97, 3.82)
Model 3 + All potential mediators	2.09^*^ (1.10, 3.95)	0.83 (0.53, 1.29)	1.69 (0.82, 3.50)

CVD mortality			
*A: Adjustment for covariates*			
Model 1	1.51 (0.61, 3.77)	2.35^***^ (1.62, 3.42)	5.34^***^ (2.98, 9.58)
Model 2	1.44 (0.58, 3.56)	2.22^***^ (1.52, 3.23)	4.53^***^ (2.45, 8.37)
Model 3	1.28 (0.54, 3.07)	1.51 (1.00, 2.30)	2.82^**^ (1.46, 5.45)
*B: Adjustment for potential mediators*			
Model 3 + Depression	1.24 (0.51, 3.04)	1.49 (0.98, 2.26)	2.62^**^ (1.34, 5.14)
Model 3 + Walking disability	1.07 (0.45, 2.53)	1.28 (0.83, 1.97)	2.23^*^ (1.14, 4.38)
Model 3 + Social participation	1.27 (0.53, 3.07)	1.44 (0.94, 2.20)	2.71^**^ (1.43, 5.17)
Model 3 + All potential mediators	1.05 (0.44, 2.54)	1.23 (0.79, 1.90)	2.10^*^ (1.07, 4.11)

A Cox proportional hazards model was applied. In Model 1, age and sex were adjusted; in Model 2, the other demographic factors (education years, the living area, income level, marital status, and primary occupation) were additionally adjusted; and in Model 3, health statuses and health behaviors (self-rated health, self-reported histories of cancer, stroke, heart disease, diabetes, dyslipidemia, and hypertension, body mass index, smoking status, exercise habits, alcohol consumption, and dietary patterns) were additionally adjusted. In Panel B, the indicators of walking ability, depression, and social participation were separately or jointly adjusted as potential mediators. The reference group was participants without hearing loss or visual loss.

^*^P < 0.05. ^**^P < 0.01. ^***^P < 0.001.

eTable 2 and eTable 3 report the results of the repeated analyses using the listwise deletion sample. The point estimates were farther than 1 compared to the main analyses, but the statistical significances were similar. eTable 4 and eTable 5 report the results of the repeated analyses using a strict definition of sensory loss. The statistical significances shown in the main analyses disappeared, but the trends were quite similar. Contrary to our expectations, the results of the analyses that separately included HL/VL in model 3 were quite similar to the main results (eTable 6).

## DISCUSSION

Self-reported HL and DSL were associated with increased all-cause mortality after adjustment for potential known confounders, including health status and comorbidity among middle-aged and older adults in Japan. In contrast, no associations between self-reported VL and all-cause mortality were observed after controlling for confounders. A synergistic effect of HL and VL on all-cause mortality was not observed. Our findings suggest that self-reported HL may be an independent risk factor of early death.

In testing the association between sensory loss and mortality, adjusting for health status including CVD risk factors and the other sensory comorbidity is considered as important to address residual confounding.^13^ In this context, previous studies reported that DSL was associated with increased all-cause mortality^14^^,^^15^ and CVD mortality^15^ and that VL was not significantly associated with all-cause mortality,^13^^–^^15^ which is consistent with our findings. On the other hand, HL was reported not to be significantly associated with all-cause mortality after adjustment of confounders,^13^^–^^15^ which is at odds with our findings. This discrepancy may be because the definition of HL in prior studies corresponded to mild hearing impairment (trouble in understanding soft speech) following World Health Organization (WHO) criteria, but our definition of HL (difficulty in understanding normal conversation) corresponded to moderate or severe levels of impairment.^40^ Also, the findings of our study were in contrast to the previous studies in Japan, where they failed to find a significant association of HL with all-cause mortality or dependence in ADL.^34^^,^^41^ This difference may be because the participants in our study included middle-aged population as well as older population and were not selected by baseline criteria, such as functional independence and long-term care insurance eligibility.

Two hypotheses may explain the association of sensory loss with mortality. First, the association may be explained by residual confounding caused by risk factors for both sensory loss and premature mortality. Although as many potential confounders as possible (including CVD risk factors) were controlled for, the assessment of behavioral risk factors was relatively crude (eg, we did not control for intensity of smoking/daily exercise or detailed dietary composition). Additional unobserved factors include noise exposure,^29^^,^^42^ neighborhood characteristics,^43^ genetic predisposition,^25^ and chronic inflammation.^44^ Second, sensory loss may cause early death via mediators, such as social isolation,^45^ deterioration in physical activity,^1^ worsened psychological health,^3^ and declining cognitive function.^1^ The association between HL and mortality has been previously reported to be partly mediated by psychological health, and social relationships, gait speed, and cognition.^10^ Karpa et al showed the effect of HL on mortality was mediated by cognitive impairment and walking ability using structural equation modeling.^7^ Our study also suggested that walking disability might explain a moderate part of the total effect of DSL on all-cause mortality, but the three potential mediators (depression, walking disability, and social participation) explained a modest portion of the association between HL/DSL and all-cause mortality. However, as information on cognitive ability was unavailable in our study, we could not infer to what degree cognitive decline as a mediator explained the association between sensory loss and mortality.

The association of HL (but not VL) with cancer mortality raises the potential for additional pathways that merit attention in future studies. For instance, in screening or treatment of cancer, hearing ability may be a critical factor in predicting the success of doctor-patient communication, and subsequent patient adherence to therapy.^46^ Anecdotally, HL is a more critical factor in doctor-patient communication than vision loss.

Some limitations should be noted. First, our use of self-reported HL or VL hampers direct comparison with the results of prior studies using audiometry and visual acuity testing. The age-specific prevalence of self-reported HL among this study’s participants (eFigure 1) was roughly comparable to that of moderate or severe hearing impairment (PTA of more than 40 dB) based on WHO criteria in a general East Asian population.^40^^,^^47^ Meanwhile, the age-specific prevalence of self-reported VL among this study’s participants was higher than that of “distance” vision impairments based on U.S. criteria in a general Japanese population.^48^ This difference might be because the definition of VL used in the main analyses of this study included “near” vision impairments, such as presbyopia,^49^ or relatively less severe vision impairment. This lenient definition might be one reason for the lack of significant association between VL and mortality observed in the main analyses, though no significant association between VL and mortality was observed even when we used the stricter definition of VL, as shown in the sensitivity analyses. Second, the causes of HL/VL were not specified. The association of sensory loss with mortality might vary according to the underlying conditions that caused HL/VL (eg, congenital HL and acquired HL). Third, this study was conducted using a community-based cohort, and the participants might not be representative of the whole population of Japan. Fourth, the main mechanism of the association of HL/DSL with mortality remains unclear, which should be a theme of further research. Fifth, the number of cause-specific deaths might be small among the groups of HL only or DSL. The results obtained from the small number of cases might suffer from relatively imprecise estimation (with wide 95% CIs). Lastly, some participants would belong to the same household. In this case, clustered data structure should have been considered, but it was impossible because we did not have information on household identification number in this study.

Nevertheless, our study has some strengths. First, this study sheds light on the relationship between functional sensory loss and mortality focusing on self-reported sensory loss, which is convenient from a pragmatic perspective. Asking about sensory loss, especially HL in the medical interview for middle-aged and older adults, may be a promising way to evaluate patients’ risk of early death. Second, to our knowledge, this is the first study to report the association between HL and increased cancer mortality even after adjustment for potential confounders, including history of cancer and health-related behaviors. Third, this cohort study had high quality data; the rate of loss of follow-up was quite low, and the causes of death were identified using death certificates issued by medical professionals.

In conclusion, this 10-year follow-up study demonstrated that self-reported HL and DSL were associated with increased mortality after adjusting for potential confounders among middle-aged and older Japanese. Self-reported HL and DSL may be useful in assessing the risk of early death.

## APPENDIX A. SUPPLEMENTARY DATA

eTable 1. The percentage of the missing values on each item

eTable 2. The association between sensory loss status and all-cause mortality: selected sample

eTable 3. The association between sensory loss status and cause-specific mortality: selected sample

eTable 4. The association between sensory loss status and all-cause mortality: strict definition of sensory loss

eTable 5. The association between sensory loss status and cause-specific mortality: strict definition of sensory loss

eTable 6. The association between sensory loss and mortality: separately adjusted model

eFigure 1. The prevalence of sensory loss. The dark color bar shows the prevalence of those who had self-reported hearing loss (Panel A) or visual loss (Panel B) in this study. The light color bar in Panel A shows the prevalence of those who had moderate or severer hearing impairment based on the WHO criteria reported in Kim et al., 2000; The light color bar in Panel B shows the prevalence of those who had vision impairment or blindness based on the US criteria reported in Yamada et al., 2010.

eAppendix 1. Mediation analyses
