# Supplementary material for: Self-Reported Hearing/Visual Loss and Mortality in Middle-Aged and Older Adults: Findings From the Komo-Ise Cohort, Japan
Source: J Epidemiol. 2020 Feb 5;30(2):67–73. doi: 10.2188/jea.JE20180198 (PMC6949181; doi:10.2188/jea.JE20180198)
Supplement: Supplementary file 1 [file je-30-067-s001.pdf]

eTable 1. The percentage of the missing values on each item

Characteristics	The percentage of missing values (%) [a]
Hearing loss only	6.4
Visual loss only	5.9
Dual sensory loss	6.7
Education level	3.4
Income level	3.6
Living area	0
Marital status	3.3
Primary occupation	5.2
Exercise habits	11.0
Alcohol consumption	2.9
Smoking status	8.1
Obesity	3.4
Preference for salty foods	1.7
Preference for sweet foods	1.7
Preference for fatty foods	2.6
Fair or better self-rated health	1.2
History of cancer	6.1
History of stroke	6.1
History of heart disease	7.7
History of diabetes	5.8
History of dyslipidemia	7.1
History of hypertension	5.9
Depression	15.2
Walking disability	5.5

[a] For each item, the number of the participants who had a missing value was divided by the number of the total analytic participants (n=9,522).

eTable 2. The association between sensory loss status and all-cause mortality: selected sample

	Hazard ratios (95% confidence intervals)			
	No hearing/visual loss	Hearing loss only	Visual loss only	Dual sensory loss
Number of participants	5816	54	435	44
Number of deaths	561	15	69	17
A: Adjustment for covariates				
Model 1	ref	1.94* (1.14, 3.28)	1.62*** (1.25, 2.09)	3.27*** (1.96, 5.47)
Model 2	ref	1.97* (1.16, 3.35)	1.56*** (1.20, 2.02)	3.28*** (1.98, 5.43)
Model 3	ref	2.23** (1.32, 3.78)	1.14 (0.87, 1.49)	1.63 (0.90, 2.96)
B: Adjustment for potential mediators				
Model 3 + Depression	ref	2.18** (1.28, 3.69)	1.14 (0.86, 1.49)	1.59 (0.88, 2.86)
Model 3 + Walking disability	ref	2.25** (1.35, 3.73)	1.01 (0.77, 1.34)	1.26 (0.66, 2.39)
Model 3 + Social participation	ref	2.20** (1.31, 3.70)	1.10 (0.83, 1.44)	1.63 (0.91, 2.91)
Model 3 + All potential mediators	ref	2.19** (1.32, 3.66)	0.99 (0.75, 1.30)	1.26 (0.68, 2.35)

A Cox proportional hazards model was applied. In Model 1, age and sex were adjusted; in Model 2, the other demographic factors (education years, marital status, the living area, income level, and primary occupation) were additionally adjusted; and in Model 3, health statuses and health behaviors (self-rated health, self-reported histories of cancer, stroke, heart disease, diabetes, dyslipidemia, and hypertension, body mass index, smoking status, exercise habits, alcohol consumption, and dietary patterns) were additionally adjusted. In Panel B, the indicators of walking ability, depression, and social participation were separately or jointly adjusted as potential mediators. The reference group was participants without hearing loss or visual loss.

* $p<0.05$. ** $p<0.01$. *** $p<0.001$.

eTable 3. The association between sensory loss status and cause-specific mortality: selected sample

	Hazard ratios (95% confidence intervals)			
	No hearing/visual loss	Hearing loss only	Visual loss only	Dual sensory loss
Number of participants	5816	54	435	44
Cancer mortality				
Number of events	201	6	20	6
A: Adjustment for covariates				
Model 1	ref	2.15 (0.95, 4.88)	1.33 (0.84, 2.11)	3.27** (1.42, 7.52)
Model 2	ref	2.24 (0.96, 5.22)	1.30 (0.82, 2.06)	3.40** (1.44, 8.00)
Model 3	ref	2.59* (1.09, 6.17)	0.98 (0.61, 1.59)	2.15 (0.87, 5.28)
B: Adjustment for potential mediators				
Model 3 + Depression	ref	2.35 (0.97, 5.67)	0.98 (0.61, 1.59)	1.86 (0.75, 4.61)
Model 3 + Walking disability	ref	2.60* (1.09, 6.17)	0.94 (0.58, 1.53)	1.95 (0.77, 4.94)
Model 3 + Social networks	ref	2.59* (1.08, 6.07)	0.98 (0.60, 1.59)	2.14 (0.87, 5.25)
Model 3 + All potential mediators	ref	2.36 (0.98, 5.67)	0.95 (0.58, 1.55)	1.71 (0.67, 4.39)
CVD mortality				
Number of events	100	1	25	8
A: Adjustment for covariates				
Model 1	ref	0.71 (0.10, 5.09)	3.16*** (2.04, 4.92)	8.12*** (3.91, 16.9)
Model 2	ref	0.70 (0.10, 4.96)	3.09*** (1.99, 4.80)	6.94*** (3.25, 14.8)
Model 3	ref	0.73 (0.11, 4.64)	2.25*** (1.38, 3.66)	3.11* (1.13, 8.58)
B: Adjustment for potential mediators				
Model 3 + Depression	ref	0.70 (0.11, 4.50)	2.24*** (1.38, 3.65)	3.04* (1.12, 8.22)
Model 3 + Walking disability	ref	0.62 (0.13, 3.04)	1.72* (1.03, 2.86)	2.19 (0.80, 5.98)
Model 3 + Social networks	ref	0.71 (0.11, 4.58)	2.09** (1.27, 3.45)	3.09* (1.17, 8.18)
Model 3 + All potential mediators	ref	0.61 (0.12, 3.13)	1.65 (0.99, 2.77)	2.22 (0.84, 5.84)

A Cox proportional hazards model was applied. In Model 1, age and sex were adjusted; in Model 2, the other demographic factors (education years, marital status, the living area, income level, marital status, and primary occupation) were additionally adjusted; and in Model 3, health statuses and health behaviors (self-rated health, self-reported histories of cancer, stroke, heart disease, diabetes, dyslipidemia, and hypertension, body mass index, smoking status, exercise habits, alcohol consumption, and dietary patterns) were additionally adjusted. In Panel B, the indicators of walking ability, depression, and social participation were separately or jointly adjusted as potential mediators. The reference group was participants without hearing loss or visual loss.

* $p<0.05$. ** $p<0.01$. *** $p<0.001$.

eTable 4. The association between sensory loss status and all-cause mortality: strict definition of sensory loss

	Hazard ratios (95% confidence intervals)		
	Hearing loss only	Visual loss only	Dual sensory loss
A: Adjustment for covariates			
Model 1	1.87 (0.98, 3.57)	1.89*** (1.42, 2.53)	3.31*** (1.76, 6.24)
Model 2	1.87 (0.99, 2.34)	1.78*** (1.35, 2.34)	3.17*** (1.68, 5.97)
Model 3	1.63 (0.87, 3.04)	1.21 (0.89, 1.66)	1.78 (0.97, 3.28)
B: Adjustment for potential mediators			
Model 3 + Depression	1.58 (0.84, 2.96)	1.21 (0.88, 1.66)	1.68 (0.90, 3.13)
Model 3 + Walking disability	1.47 (0.77, 2.80)	1.12 (0.82, 1.53)	1.66 (0.90, 3.05)
Model 3 + Social networks	1.62 (0.87, 3.02)	1.16 (0.84, 1.60)	1.73 (0.94, 3.17)
Model 3 + All potential mediators	1.44 (0.75, 2.75)	1.08 (0.78, 1.49)	1.56 (0.84, 2.89)

A Cox proportional hazards model was applied. In Model 1, age and sex were adjusted; in Model 2, the other demographic factors (education years, marital status, the living area, income level, and primary occupation) were additionally adjusted; and in Model 3, health statuses and health behaviors (self-rated health, self-reported histories of cancer, stroke, heart disease, diabetes, dyslipidemia, and hypertension, body mass index, smoking status, exercise habits, alcohol consumption, and dietary patterns) were additionally adjusted. In Panel B, the indicators of walking ability, depression, and social participation were separately or jointly adjusted as potential mediators. The reference group was participants without hearing loss or visual loss.

*** $p<0.001$.

eTable 5. The association between sensory loss status and cause-specific mortality: strict definition of sensory loss

	Hazard ratios (95% confidence intervals)		
	Hearing loss only	Visual loss only	Dual sensory loss
Cancer mortality			
A: Adjustment for covariates			
Model 1	2.09 (0.77, 5.72)	1.57 (0.93, 2.65)	4.60*** (2.04, 10.4)
Model 2	2.31 (0.84, 6.37)	1.52 (0.89, 2.57)	4.97*** (2.14, 11.5)
Model 3	2.40 (0.87, 6.66)	1.18 (0.69, 2.02)	3.26* (1.30, 8.16)
B: Adjustment for potential mediators			
Model 3 + Depression	2.31 (0.82, 6.51)	1.17 (0.68, 2.01)	2.95* (1.14, 7.60)
Model 3 + Walking disability	2.34 (0.84, 6.55)	1.16 (0.67, 1.99)	3.18* (1.28, 7.92)
Model 3 + Social networks	2.40 (0.87, 6.66)	1.17 (0.68, 2.02)	3.23* (1.29, 8.10)
Model 3 + All potential mediators	2.27 (0.80, 6.43)	1.15 (0.66, 1.99)	2.88* (1.12, 7.41)
CVD mortality			
A: Adjustment for covariates			
Model 1	1.81 (0.42, 7.76)	3.20*** (1.98, 5.18)	6.98*** (2.94, 16.5)
Model 2	1.55 (0.37, 6.47)	2.90*** (1.78, 4.73)	5.88*** (2.46, 14.1)
Model 3	1.22 (0.33, 4.53)	1.93* (1.12, 3.33)	2.97* (1.20, 7.38)
B: Adjustment for potential mediators			
Model 3 + Depression	1.16 (0.31, 4.27)	1.91* (1.09, 3.34)	2.73* (1.06, 7.03)
Model 3 + Walking disability	1.03 (0.27, 3.91)	1.67 (0.97, 2.85)	2.81 (1.14, 6.91)
Model 3 + Social networks	1.20 (0.33, 4.35)	1.79* (1.02, 3.13)	2.92* (1.20, 7.12)
Model 3 + All potential mediators	1.00 (0.27, 3.68)	1.58 (0.90, 2.76)	2.62* (1.04, 6.64)

A Cox proportional hazards model was applied. In Model 1, age and sex were adjusted; in Model 2, the other demographic factors (education years, marital status, the living area, income level, and primary occupation) were additionally adjusted; and in Model 3, health statuses and health behaviors (self-rated health, self-reported histories of cancer, stroke, heart disease, diabetes, dyslipidemia, and hypertension, body mass index, smoking status, exercise habits, alcohol consumption, and dietary patterns) were additionally adjusted. In Panel B, the indicators of walking ability, depression, and social participation were separately or jointly adjusted as potential mediators. The reference group was participants without hearing loss or visual loss.

* $p<0.05$. *** $p<0.001$.

eTable 6. The association between sensory loss and mortality: separately adjusted model

	Hazard ratios (95% confidence intervals)	
	Hearing loss	Visual loss
All-cause mortality	1.72** (1.18, 2.53)	1.00 (0.79, 1.27)
Cancer mortality	2.19* (1.17, 4.13)	0.81 (0.53, 1.26)
CVD mortality	1.15 (0.48, 2.76)	1.36 (0.89, 2.08)

A Cox proportional hazards model was applied. In Model 1, age and sex were adjusted; in Model 2, the other demographic factors (education years, marital status, the living area, income level, marital status, and primary occupation) were additionally adjusted; and in Model 3, health statuses and health behaviors (self-rated health, self-reported histories of cancer, stroke, heart disease, diabetes, dyslipidemia, and hypertension, body mass index, smoking status, exercise habits, alcohol consumption, and dietary patterns) were additionally adjusted. In Panel B, the indicators of walking ability, depression, and social participation were separately or jointly adjusted as potential mediators. The reference group was participants without hearing loss or visual loss.

* p<0.05. ** p<0.01.

eFigure 1. The prevalence of sensory loss. The dark color bar shows the prevalence of those who had self-reported hearing loss (Panel A) or visual loss (Panel B) in this study. The light color bar in Panel A shows the prevalence of those who had moderate or severer hearing impairment based on the WHO criteria reported in Kim et al., 2000; The light color bar in Panel B shows the prevalence of those who had vision impairment or blindness based on the US criteria reported in Yamada et al., 2010.

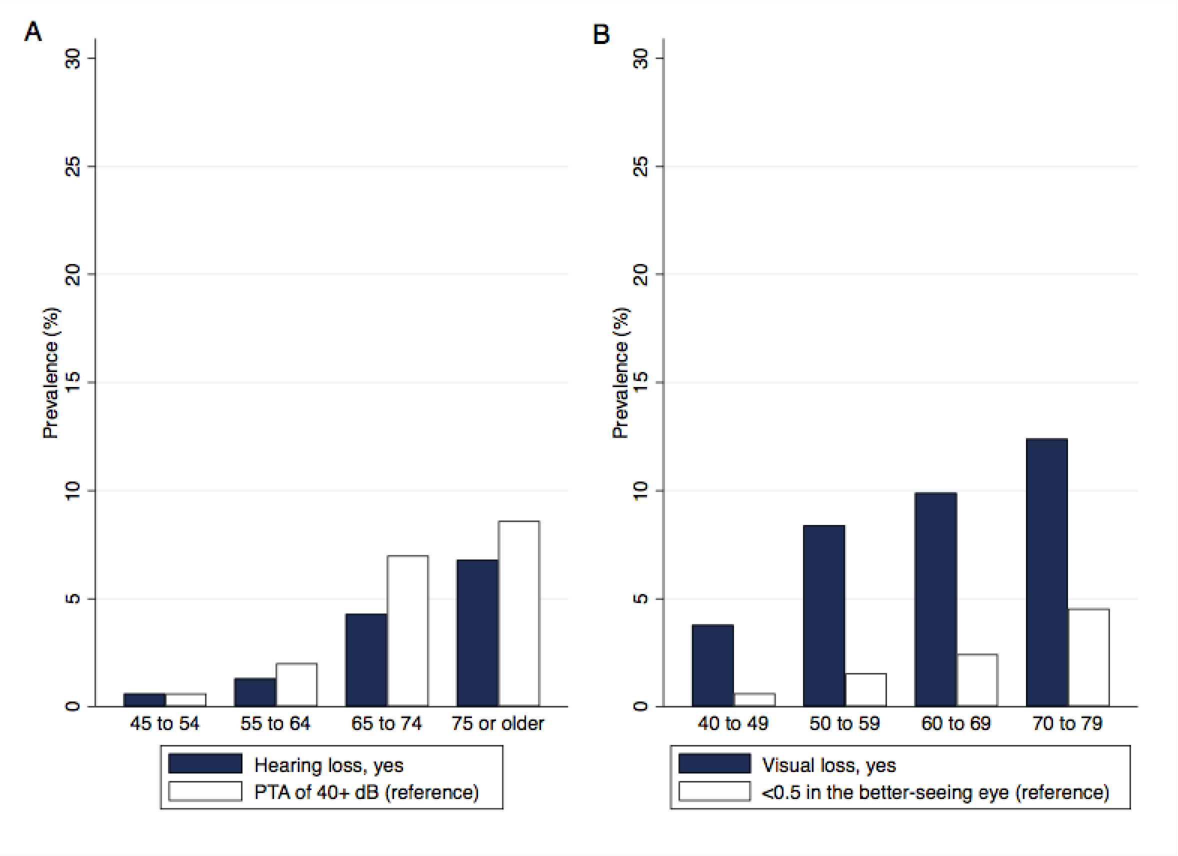

eAppendix 1. Mediation analyses

We explain how the ratio of the natural indirect effect to the total effects in the association of HL only, VL only, or DSL with mortality is estimated. This mediation analysis in the context of a survival analysis was conducted following Lange and Hansen, 2010.

Let M be a potential mediator, X be an exposure (either of HL only, VL only, or DSL), and Z be the other baseline covariates (including the sensory loss comorbidity other than the exposure). Suppose the following two equations:

(1) $\lambda(t|X,Z,M) = \beta_0 + \beta_1 X + \beta_2(t)Z + \beta_3 M + \varepsilon$

, and

(2) $M = \alpha_0 + \alpha_1 X + \alpha_2 Z + \upsilon.$

Here, $\lambda(t|X,Z,M)$ is a conditional hazard function; ε and υ are error terms, respectively. The equation (1) is called an Aalen's additive hazard model. We assume neither the exposure nor the mediator has time-dependent effects (λ1 and λ3 is time-invariant). Also, we assume there are no unmeasured confounders for the exposure-outcome, the mediator-outcome, and the exposure-mediator relations, and that there is no variable that is affected by the exposure and itself affects both the mediator and the outcome (each mediator does not affect any other mediators).

By plugging (2) into (1), the conditional hazard function is written as follows:

$$\lambda(t|X,Z) = \beta_0 + \beta_1 X + \beta_2(t)Z + \beta_3(\alpha_0 + \alpha_1 X + \alpha_2 Z + \upsilon) + \varepsilon$$

$$= \beta_0 + \beta_3\alpha_0 + (\beta_1 + \beta_3\alpha_1)X + (\beta_2(t) + \beta_3\alpha_2)Z + \beta_3\upsilon + \varepsilon$$

The right side indicates that the total effect of X on hazard is $\beta_1 + \beta_3\alpha_1$. Also, the right side of the equation (1) indicates the natural direct effect of X on hazard is β_1. Thus, the

natural indirect effect of X via a mediator M on hazard is $\beta_3\alpha_1$, and the ratio of the natural indirect effect to the total effect was $\frac{\beta_3\alpha_1}{\beta_1+\beta_3\alpha_1}$.

The command “timereg” in software R (https://cran.r-project.org/web/packages/timereg/timereg.pdf) was used to estimate β_1 and β_3 in the equation (1). Ordinary least squares estimation was applied to estimate α_1 in the equation (2). We also estimated 95% confidence intervals following the eAppendix of Lange and Hansen, 2010.
